# Changes in Rx1 and Pax6 activity at eye field stages differentially alter the production of amacrine neurotransmitter subtypes in *Xenopus*

**Published:** 2007-01-26

**Authors:** Norann A. Zaghloul, Sally A. Moody

**Affiliations:** Department of Anatomy and Cell Biology, Program in Genetics, Institute for Biomedical Sciences, The George Washington University, Washington, DC

## Abstract

**Purpose:**

Both *rx1* and *pax6* are expressed during the initial formation of the vertebrate eye field, and they are thought to be crucial for maintenance of the retinal stem cells in the ciliary marginal zone. However, both genes continue to be expressed in different layers of the differentiating retina, suggesting that they have additional roles in cell type specification. Because previous work suggested that amacrine cell subtypes are derived from biased progenitors in the eye field, we tested whether altering Rx1 or Pax6 activity during eye field stages affects the production of three neurotransmitter subtypes of amacrine cells.

**Methods:**

Gain-of-function and loss-of-function hormone-inducible constructs of Rx1 and Pax6 were used to alter Rx1 and Pax6 protein or activity levels after the formation of the eye field. The major-retina producing blastomere of the 32-cell stage *Xenopus* embryo (D1.1.1) was injected with mRNA encoding one of these proteins and mRNA encoding GFP to label the altered lineage. Embryos were treated with synthetic hormone at either early (stage 12) or late (stage 16) eye field stages and they developed to tadpole stages (stage 44/45) when the cells in the central retina have differentiated. Amacrine cell subtypes (dopamine [DA], neuropeptide Y [NPY], γ aminobutyrate acid [GABA]) were detected by immunofluorescence histology and the numbers of each type of cell produced within the affected lineage were counted. The percent contribution of the D1.1.1 lineage to a particular amacrine subtype after stage 12 or stage 16 hormone treatment were independently compared to those from *gfp* mRNA-injected control embryos that were similarly treated with hormone.

**Results:**

Increasing Rx1 at early eye field stages promotes NPY amacrine cells and represses GABA and DA amacrine cells, and at late eye field stages significantly represses DA and NPY phenotypes but has a diminished effect on the GABA phenotype. Increasing Pax6 at early eye field stages represses NPY and DA amacrine cells but does not affect the GABA phenotype, whereas in the late eye field it significantly represses only the DA phenotype.

**Conclusions:**

Rx1 and Pax6 differentially modify the ability of eye field precursors to produce different neurotransmitter subtypes of amacrine cells. These effects varied for each of the subtypes investigated, indicating that amacrine cells are not all specified by a single genetic program. Furthermore, some cases were time-dependent, indicating that the downstream effects change as development proceeds.

## Introduction

The vertebrate neural retina is comprised of seven major cell types organized into defined layers, all of which are derived from the eye field, a subpopulation of cells in the anterior neural plate [1,2]. The eye field is defined by the overlapping expression of several transcription factors that are thought to functionally define those cells that give rise to the neural retina, sometimes referred to as the most primitive retinal stem cells [3-6]. The earliest expressed eye field transcription factors, *rx1* and *pax6* are often termed "master" regulatory genes of eye development because knock-out studies demonstrate that each is necessary for eye formation, and over-expression studies show that each is sufficient to produce ectopic eye tissue [7-14]. Consistent with this idea, both genes continue to be expressed throughout the regions that contain the retinal stem and progenitor cells, i.e., the optic vesicle, the neural layer of the optic cup and the ciliary marginal zone (CMZ) of the differentiated retina. But in addition, both are expressed in specific layers of the retina as the neurons differentiate, suggesting that they may have later roles in defining different cell types. Studies of *rx1* are not as extensive as those of*pax6* although the two genes share many similarities in function [15]. In *Xenopus*, *rx1* is expressed in the outer nuclear layer (ONL; rod and cone photoreceptors) and the outer zone of the inner nuclear layer (OINL; horizontal, bipolar and Müller glial cells) and *pax6* is expressed in the ganglion cell layer (GCL; ganglion cells) and inner zone of the INL (IINL; amacrine cells) [2,16,17].

It has been difficult to assess the later roles of *rx1* and *pax6* because both are required for establishing the eye field. Recently, a conditional knock-out of *pax6* in the mouse CMZ demonstrated that *pax6* is necessary for continued production of all retinal cell types except amacrine cells [18], but there has been no similar report to date for *rx1* function. We are particularly interested in identifying whether *rx1* or *pax6* differentially impact amacrine cell fate because previous work showed that *Xenopus* retinal precursors have different developmental potentials to produce subtypes of amacrine cells [6]. Early embryonic blastomere precursors are differentially biased towards producing dopamine (DA), neuropeptide Y (NPY) and serotonin (5HT) amacrine cells [19,20], whereas there is no bias to produce GABA or glycine amacrine cells [21]. Labeling of single cells in the eye field demonstrated that about half of these cells are multipotent, producing cell types in all layers, and about half are biased towards INL fates, in particular amacrine cells [22]. Sampling of all quadrants of the eye field and both deep and superficial ectodermal layers produced both types of progenitors (multipotent and biased), indicating that they are intermixed throughout the eye field. Furthermore, both DA and NPY amacrine cells, but not 5HT amacrine cells, appear to be lineage restricted during eye field stages [22].

These observations suggest that genes expressed in the eye field may differentially influence the specification of retinal progenitors that give rise to different sets of differentiated cell types. Amacrine cells are well known to be a diverse population of interneurons; numerous different types have been defined by neurotransmitter expression alone [23]. Because we have quantified the number of several neurotransmitter subtypes of amacrine cells that descend from defined embryonic lineages [19-21], we tested whether altered Rx1 or Pax6 levels or activity affects the production of three neurotransmitter subtypes of amacrine cells. To avoid affecting the initial establishment of the eye field, we utilized hormone-inducible mRNA constructs that could be activated after eye field formation by synthetic hormone treatment [24,25], and we targeted these constructs to a single embryonic blastomere lineage whose specific contribution to each amacrine cell type can be determined. We find that increased Rx1 levels in the early eye field promotes NPY amacrine cells and represses GABA and DA amacrine cells, and in the late eye field significantly represses DA and NPY phenotypes but has a diminished effect on the GABA phenotype. Increased Pax6 levels in the early eye field represses NPY and DA amacrine cells but does not affect the GABA phenotype, and in the late eye field significantly represses only the DA phenotype. These results indicate that altered levels of Rx1 and Pax6 differentially modify the ability of the eye field precursors to produce different neurotransmitter subtypes of amacrine cells. Importantly, these effects varied for each of the subtypes investigated, indicating that amacrine cells are not all specified by a single genetic program. Furthermore, some cases were time-dependent, indicating that the downstream effects change as development proceeds.

## Methods

### Generation and collection of embryos

**Figure 1:**
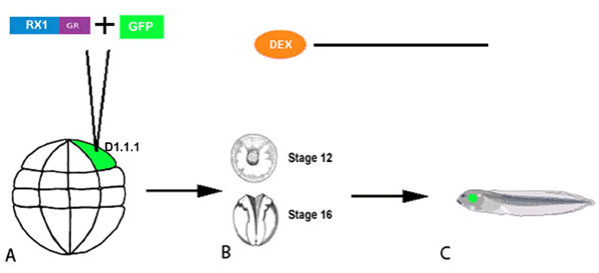
Blastomere injection and activation of gene expression. **A**: The major blastomere progenitor of the retina (D1.1.1) in a 32-cell stage embryo was injected with mRNAs encoding hormone-inducible *rx1*or *pax6* constructs and *gfp* as a lineage tracer. **B**: Exogenous gene expression was induced in early (Stage 12) or late (Stage 16) eye fields by incubation in dexamethasone solution. Hormone treatment was continued throughout the culture period. (C) Embryos were raised to tadpole stages (stage 44/45) when amacrine cell subtypes have differentiated and can be labeled with neurotransmitter-specific antibodies.

Fertilized *Xenopus laevis* embryos were obtained from adult frogs induced to mate after injection of human chorionic gonadotropin. After chemical removal of the jelly coat, embryos were selected as previously described [26] to allow the major blastomere precursor of the retina (blastomere D1.1.1; Figure 1) to be identified [27].

### Targeted microinjection of synthetic mRNAs

For gain-of-function studies, the open reading frames of *rx1* [10] and *pax6* [16] were fused by PCR to the ligand-binding domain of the human glucocorticoid receptor (GR) as described [25]. For loss-of-function studies, the same was done with previously characterized *rx1* Engrailed repressor (*rx1EnR* [28]; ) and dominant-negative *pax6* (*dnpax6*) [11] constructs. Capped, polyadenylated mRNAs were synthesized in vitro (Ambion, Inc.), and mixed with *green fluorescent protein* (*gfp*; 100 pg) mRNA as a lineage tracer. Each experimental mRNA (*rx1-GR*, 50 pg; *pax6-GR*, 50 pg; *rx1EnR-GR*, 400 pg; *dnpax6-GR*, 60 pg) was microinjected into blastomere D1.1.1 (Figure 1). The amount of test mRNA for injection was determined in previous publications to: (1) effectively induce ectopic retinal phenotypes with wild type constructs [10,11,13]; (2) effectively reduce downstream target gene expression (engrailed repressor construct) or endogenous protein activity (dominant-negative construct) [11,28]; and (3) produce no signs of cell toxicity [13]. Each experiment was repeated at least three times.

Exogenous gene expression was targeted to the D1.1.1 blastomere, which produces about 50% of the differentiated cells in the tadpole retina [27], in order to avoid a global effect on the eye field that might perturb later retinogenesis. Previous lineage studies showed that the descendants of the D1.1.1 blastomere are scattered throughout the eye field, intermixed with cells derived from adjacent blastomeres [27,29]. In addition, this blastomere produces a defined number of DA, NPY and GABA amacrine cells [19,21], which allows one to precisely quantify cell phenotype changes resulting from alterations in transcription factor level/activity.

After mRNA injection, the cells synthesize the fusion proteins, but the GR domain forms a complex with endogenous heat shock proteins that prevents the transcription factor from entering the nucleus [24,25]. To uncouple this complex and allow nuclear translocation, control and injected embryos were incubated in synthetic hormone (10 mM dexamethasone) according to published protocols [25]. To ensure that the GR constructs function as expected, injected embryos were treated with hormone immediately after mRNA injection; for each construct these embryos phenocopied those injected with the non-GR versions, i.e., wild-type mRNAs [10,11,16,28]. For the experiments reported herein, embryos were treated with hormone starting at two different time points in eye field development (stage 12, early neural plate; stage 16, neural fold) [30]; hormone treatment was maintained in the medium throughout the culture period (Figure 1). Experiments in tissue culture and in whole embryos with similar GR-fusion constructs indicate that robust protein activation occurs rapidly (within 90 min) after hormone treatment, and is maintained for several days [24,25,31,32]. Therefore, we assume that the GR-fusion proteins are available to affect downstream targets throughout the culture period of our experiments. Some embryos were injected with the GR-constructs and raised in the absence of hormone; DA amacrine cell numbers were unchanged from controls indicating that the GR-constructs have no effects in the absence of hormone, in accord with published accounts [24,25,31,32]. Control embryos were injected with only *gfp* mRNA and treated with dexamethasone at eye field stages; amacrine subtype cell counts from these embryos were identical to those from *gfp* mRNA-injected embryos not exposed to hormone [19,21], indicating that hormone treatment alone does not affect amacrine cell fates.

### Immunostaining and cell counting

Embryos were raised in the continuous presence of hormone to stage 44/45, fixed in 4% paraformaldehyde solution (MEMPFA [33]; ) and scanned for GFP expression to analyze only embryos in which exogenous gene expression was successful. Embryos were cryoprotected overnight at 4 °C in 30% sucrose/0.1 M phosphate buffer solution, embedded in Tissue-Tek OCT medium (Miles, Inc.), and cut serially (14 mm) with a cryostat. Immunostaining was carried out as previously described [19] using mouse anti-tyrosine hydroxylase (1:200; Immunostar) to detect DA amacrine cells, rabbit anti-GABA (1:500; Immunostar) to detect GABA amacrine cells and rabbit anti-NPY (1:200; Immunostar) to detect NPY amacrine cells. AlexaFluor-conjugated Texas Red goat anti-mouse IgG or goat anti-rabbit IgG secondary antibodies (Molecular Probes) were applied at a concentration of 1:200. Sections were analyzed using an epifluorescence microscope equipped with a dual pass GFP/Texas Red barrier filter set. The total number of immunolabeled cells and the number of these cells that also were GFP labeled was counted in every section through the retina (about 35-40 sections per retina); positive cells were confirmed by focusing through the entire thickness of the section with single pass Texas Red and single pass GFP barrier filters. Fluorescent images were collected with a BioRad MRC1024 laser scanning confocal microscope.

The proportion of immuno-positive cells that were also labeled with GFP (i.e., derived from the mRNA-injected lineage) was calculated. Percent contributions of the D1.1.1 lineage to a particular amacrine subtype after stage 12 induction or stage16 induction were independently compared to those from *gfp* mRNA-injected control embryos that were similarly treated with dexamethasone (n=10 embryos per data set) by the Student's unpaired t-test (<0.05); each treatment data set passed the equal variance test. The Student's unpaired t-test also was used to determine if there is a significant difference between the stage 12 and stage 16 induction data sets for each neurotransmitter/injected mRNA group.

## Results

### Altering Rx1 level/activity at eye field stages differentially affects amacrine subtypes

**Figure 2:**
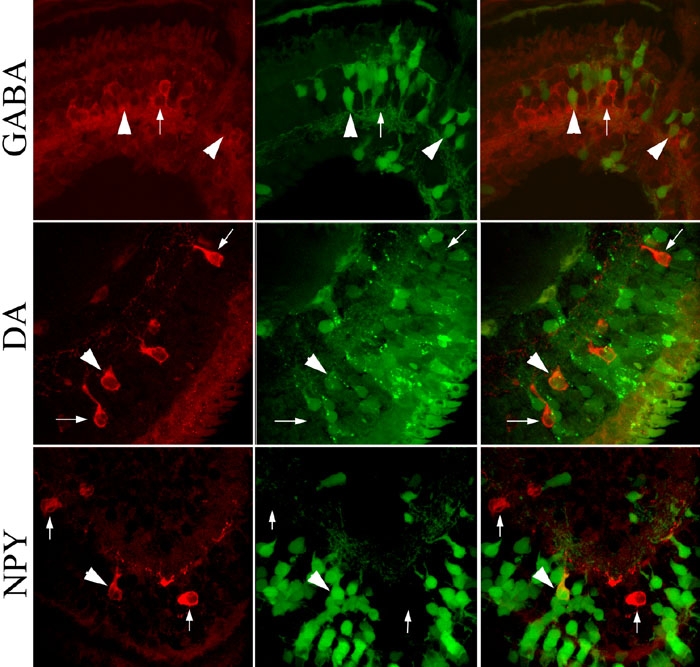
Labeling of amacrine cell subtypes. Sections of retina were labeled with antibodies to distinguish amacrine subtypes (red cells); those descended from the injected blastomere express GFP (green cells). Top row: Large numbers of amacrine cells express GABA (left panel). Large arrowheads indicate two GABA amacrine cells descended from D1.1.1 blastomere (green in middle panel and double-labeled in merged right panel). Small arrow indicates a GABA amacrine cell that is not GFP-labeled. Middle row: Dopamine (DA) amacrine cells are less abundant (left panel). Large arrowhead indicates a DA amacrine cell descended from D1.1.1 blastomere (green in middle panel and double-labeled in merged right panel). Small arrows indicate two DA amacrine cells that are not GFP-labeled. Bottom row: NPY amacrine cells also are less abundant (left panel). Large arrowhead indicates a NPY amacrine cell descended from D1.1.1 blastomere (green in middle panel and double-labeled in merged right panel). Small arrows indicate two NPY amacrine cells that are not GFP-labeled. Each image was collected with 40x oil lens, zoom set at 1.9, in a 1024x1024 pixel field, and pixel size equal to 0.12 μm.

**Figure 3:**
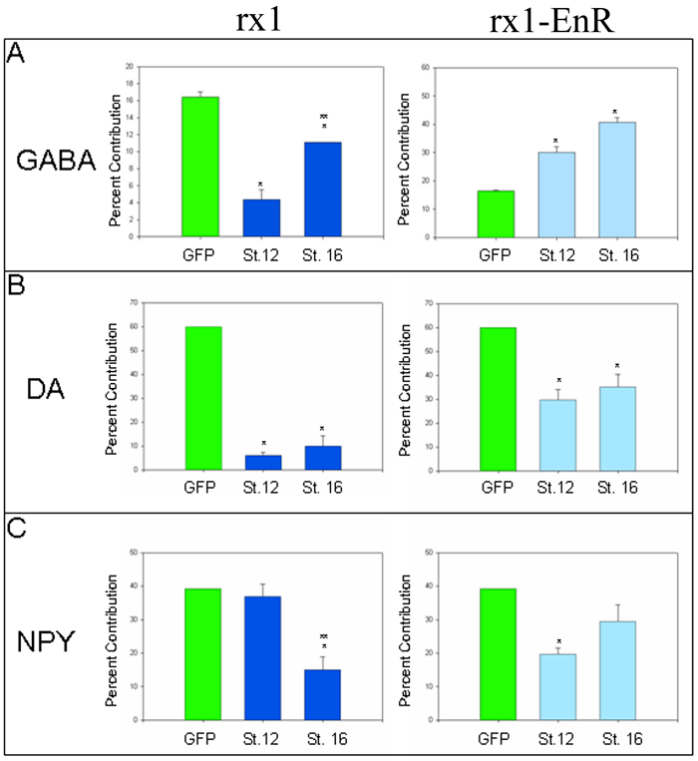
Altered Rx1 levels/activity affect all three amacrine cell subtypes. *rx1* gain-of-function (dark blue bars) and loss-of function (*rx1-EnR*; light blue bars) were induced at early (St. 12) or late (St. 16) eye field stages. The percent contribution that the injected blastomere lineage made to the total number of the subtype was determined for: (**A**) GABA, (**B**) DA and (**C**) NPY amacrine cells. Bars indicate SEM. A single asterisk (*) indicates a significant difference (p<0.05) compared to *gfp* mRNA-injected control embryos that also were treated with dexamethasone (green bars). A double asterisk (**) over a stage 16 bar indicates a significant difference (p<0.05) compared to stage 12 induction data. All samples passed the equal variance test. *rx1* gain-of-function at both eye field stages caused a significant reduction of GABA and DA amacrine cells, but the GABA reduction was significantly less at stage 16. In contrast, NPY amacrine cells were reduced only at the late stage. *rx1* loss-of-function increased GABA cell production and reduced DA amacrine cell production equivalently at both eye field stages; it significantly decreased NPY amacrine cells only at stage 12.

It has been well-established that *rx1* is both necessary and sufficient to establish the eye field and it is proposed to maintain the retinal stem cells of the optic cup, vesicle and CMZ. Its later expression in the ONL and OINL of the layered retina suggests that it may additionally repress the production of amacrine cells, which reside in the IINL. We tested whether increasing the level of Rx1 in the eye field, when biased INL progenitors have been identified [22], affects the production of three neurotransmitter subtypes of amacrine cells. In *gfp* mRNA-injected, hormone-treated control embryos the D1.1.1 lineage produces about 16% of GABA amacrine cells (Figure 2, Figure 3A). Increasing Rx1 levels in this lineage by injection of *rx1-GR* mRNA and subsequent hormone treatment beginning at stage 12 significantly repressed this phenotype; this reduction was detectable but significantly less dramatic after hormone treatment at stage 16 (Figure 3A), indicating a window of sensitivity to increased levels of Rx1 during early eye field stages. Consistent with these results that indicate that Rx1 negatively regulates the production of GABA amacrine cells, decreasing Rx1 target gene activation by injection of a repressive *rx1* construct (*rx1EnR-GR* mRNA) and subsequent hormone treatment beginning at either eye field stage significantly increased GABA amacrine cell numbers (Figure 3A). In *gfp* mRNA-injected, hormone-treated control embryos the D1.1.1 lineage produces about 60% of DA amacrine cells (Figure 2, Figure 3B). Increasing Rx1 levels beginning at either stage 12 or 16 significantly repressed the D1.1.1 lineage contribution to DA amacrine cells (Figure 3B). Decreasing Rx1 target gene activation beginning at either stage also reduced DA amacrine cell numbers (Figure 3B), but the effect was much smaller than that resulting from Rx1 gain-of-function. In *gfp* mRNA-injected, hormone-treated control embryos the D1.1.1 lineage produces about 40% of the rare NPY amacrine cells (Figure 2, Figure 3C). Increasing Rx1 levels in this lineage beginning at stage 12 did not affect NPY amacrine cell numbers, whereas hormone treatment at stage 16 caused a significant reduction. Conversely, decreasing Rx1 target gene activation repressed NPY amacrine cells when activated by hormone treatment beginning at stage 12, but did not have a significant effect when activated at stage 16. Thus, an early high level of Rx1 is required for NPY amacrine cell production, whereas a later high level of Rx1 represses it.

These data demonstrate that the levels of Rx1 and target gene activation have differential effects on these three neurotransmitter subtypes of amacrine cells, and that these effects are time-dependent. Increasing Rx1 levels beginning in the early eye field allows NPY amacrine cells to form in normal numbers and represses GABA and DA amacrine cells, whereas increasing Rx1 levels beginning in the late eye field significantly represses DA and NPY phenotypes but has a diminished effect on the GABA phenotype.

### Altering Pax6 level/activity at eye field stages alters a different subset of amacrine subtypes

**Figure 4:**
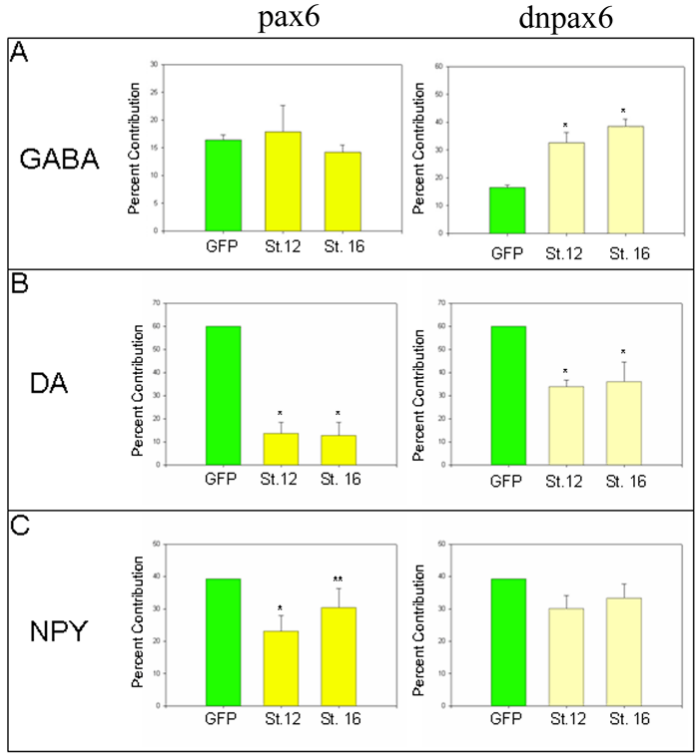
Altered Pax6 levels/activity differentially affect amacrine cell subtypes. *pax6* gain-of-function (dark yellow bars) and loss-of-function (*dnpax6*, light yellow bars) were induced at early (St. 12) and late (St. 16) eye field stages, and analyzed as in Figure 3. *pax6* gain-of-function at either eye field stage significantly reduced the D1.1.1 contribution to DA amacrine cells, whereas it only affected NPY cells at stage 12. *pax6* loss-of-function at both stages significantly increased GABA cells and significantly decreased DA cells. NPY amacrine cells were not significantly affected by *pax6* loss-of-function at either stage.

**Figure 5:**
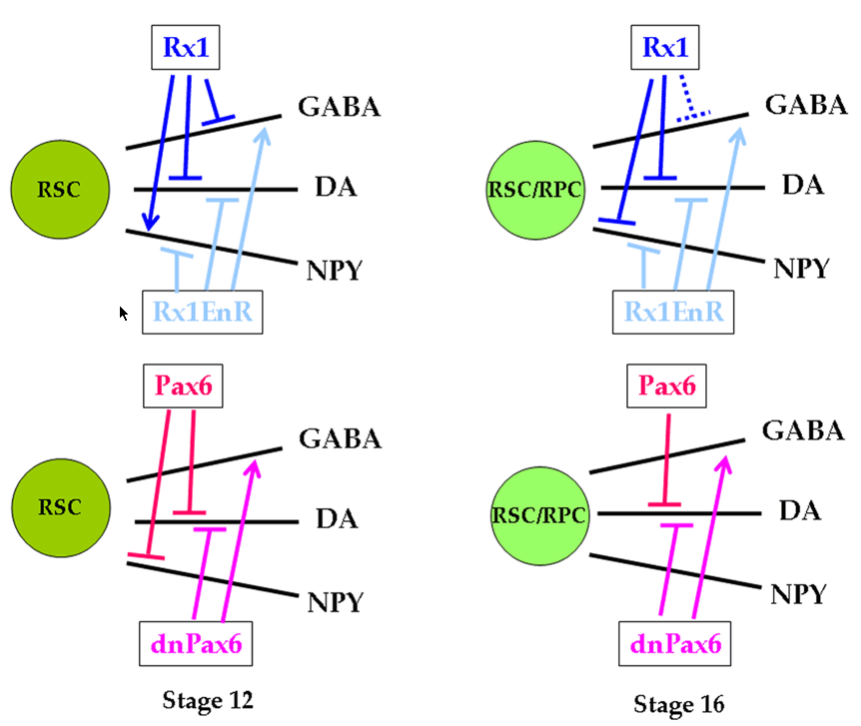
The developmental programs that produce amacrine cell subtypes are differentially affected by Rx1 and Pax6 in a time-dependent manner. The effects on the number of amacrine cells produced after induction of the different Rx1 and Pax6 constructs at the two eye field stages are summarized. Retinal stem cells (RSC) in the stage 12 eye field are repressed by Rx1 from producing GABA and DA amacrine cells, but Rx1 is required for NPY amacrine cells. In contrast, Pax6 represses the production of DA and NPY amacrine cells. Loss-of-function of either Rx1 (by Rx1EnR) or Pax6 (by dnPax6) is required for GABA amacrine cell production. In the stage 16 eye field, which is likely comprised of both RSC and retinal progenitor cells (RPC) [22], Rx1 continues to repress DA amacrine cells, but the effect on GABA amacrine cells is much reduced. In addition, Rx1 now represses NPY amacrine cells. Pax6 continues to repress DA cells, but the effect on NPY cells is no longer detectable. As at stage 12, loss-of-function of either Rx1 (by Rx1EnR) or Pax6 (by dnPax6) is required for GABA amacrine cell production. These data indicate that Rx1 and Pax6 differentially affect the production of the different amacrine subtypes over time. Because both genes are considered to be transcriptional activators [28,43], their inhibitory effects on amacrine subtypes are likely to be transcriptionally indirect.

*pax6* also is both necessary and sufficient to establish the eye field and is proposed to specify the retinal stem/progenitor cells of the optic cup, vesicle and CMZ. Although a conditional knock-out of *pax6* indicates that this gene is not required for amacrine cell genesis [18], its later expression in the IINL suggests that it may promote the differentiation of some amacrine cell subtypes. Increasing Pax6 levels in the D1.1.1 lineage by injection of *pax6-GR* mRNA and subsequent hormone treatment beginning at either eye field stage had no significant effect on GABA amacrine cell numbers, whereas decreasing Pax6 activity by injection of a dominant-negative construct (*dnpax6-GR* mRNA) significantly increased GABA amacrine cells (Figure 4A). Increasing Pax6 levels beginning at either eye field stage significantly decreased the numbers of DA amacrine cells in the D1.1.1 lineage; decreasing Pax6 activity at either stage also repressed DA amacrine cells but the effect was smaller than that resulting from *pax6* gain-of-function (Figure 4B). This result is similar to the effects of Rx1 levels on DA amacrine cells (Figure 3B). Increasing Pax6 levels beginning at stage 12 repressed NPY amacrine cell numbers, whereas hormone treatment at stage 16 had no significant effect (Figure 4C). Decreasing Pax6 activity at either stage had no significant effect on NPY amacrine cell numbers (Figure 4C). These data indicate that: (1) increasing Pax6 levels beginning in the early eye field represses NPY and DA amacrine cells but do not affect the GABA phenotype; (2) increasing Pax6 levels beginning in the late eye field significantly represses only the DA phenotype; (3) decreasing Pax6 activity beginning at either eye field stage increases GABA and represses DA amacrine cells; but (4) NPY cells are produced independent of reduced Pax6 activity. Thus, although both Rx1 and Pax6 are considered retinal stem cell gene products, their altered levels/activity beginning during the period of development when different INL progenitors have first been detected have differential impacts on the production of amacrine subtypes (Figure 5). Because both proteins are considered to be transcriptional activators [28,34], their inhibitory effects on amacrine subtypes are likely to be transcriptionally indirect.

## Discussion

Much work in retinal development has focused on the genetic mechanisms that specify cell types and their differentiation at developmental stages after eye cup formation [35], but little is known about how earlier events during eye development affect these processes. Both *rx1* and *pax6* are expressed during the initial formation of the eye field, and they are thought to be crucial for the production of definitive retinal stem cells because loss-of-function mutants result in severe eye defects [7,8,10,12,14]. However, both genes continue to be expressed differentially in particular layers of the differentiating retina, suggesting that they may have additional, albeit differing, roles in cell type specification. In addition, it has been demonstrated that during eye field stages when these two genes are broadly expressed in retinal stem cells, differentially biased progenitors arise that produce predominantly INL cells including amacrine cells [22]. Therefore, we sought to determine whether altering Rx1 or Pax6 levels/activity during this period affects amacrine subtype specification. We demonstrate that Rx1 or Pax6 have differential effects on the production of three different neurotransmitter subtypes of amacrine cells (Figure 5).

### Specification of amacrine cells

A large number of transcription factors affect the diversity and numbers of distinct retinal cell types [34-40]. For example, a combination of bHLH factors appears to be necessary for amacrine cell production. Over-expression of *NeuroD* in rat retinal progenitor cells results in a nearly two-fold increase in amacrine cells [41], and loss of *NeuroD* in combination with *Ath3* knock-out reduces amacrine cells [42]. Mouse triple knockouts for *Ath3, Ngn2*, and *Ash1* show a severe decrease in amacrine, horizontal and bipolar cells, of which *Ngn2* is the critical gene for the amacrine cells [43].

Much less is known about the role of homeobox-containing transcription factors in amacrine cell specification. Early studies suggested that *rx1* is not expressed in differentiated retinal cells, but more recent work indicates that in human and mouse, *rx1* is expressed in several layers of the adult retina [15]. In zebrafish, *rx1* expression is detected in cone photoreceptors of the adult eye [44], and in *Xenopus* tadpole retina, *rx1* is expressed in the ONL and OINL [2,17]. Recent evidence suggests that *rx1* promotes photoreceptor cell fate (reviewed in [45]) and Rx/Rax interacts with the photoreceptor-specific element, PCE-1, to activate its expression [46]. Embryonic stem cells expressing *rx1* produce cells having a photoreceptor phenotype when cultured in the presence of embryonic retinal cells [47].

Conditional inactivation of *pax6* in mice in the distal optic cup before the onset of differentiation results in the exclusive production of amacrine cells, suggesting that late Pax6 activity is necessary for all cell types except amacrine cells [18]. This study further showed that *pax6* is required for the expression of *Ngn2, Ath5* and *Ash1* but not for *NeuroD*, indicating that the differentiation of amacrine cells is mediated by NeuroD, in conjunction with Ath3 and perhaps other bHLH factors, independently of Pax6.

Our results provide important new information regarding amacrine cell specification by retinal transcription factors. First, we are the first to report that altered Rx1 levels significantly impact amacrine cell production. It is likely that we were able to detect these changes because we manipulated Rx1 levels after eye field formation and we monitored specific subtypes that can be identified with markers and precisely quantified due to their small numbers in the tadpole retina. Second, the effects of altering Rx1 and Pax6 levels on the three neurotransmitter subtypes were not the same, indicating that different genetic programs may independently or differentially affect amacrine subtype production. Third, Pax6 appears to differentially regulate amacrine subtypes. Our data indicate that: (1) the DA amacrine phenotype is repressed by both Rx1 and Pax6 throughout eye field stages; (2) Rx1 strongly represses the GABA phenotype whereas Pax6 does not; (3) GABA amacrine cells can differentiate independent of Pax6 and Rx1; and (4) NPY amacrine cells require Rx1 and are repressed by Pax6 at early but not at late eye field stages (Figure 5). A conditional *pax6* knock-out in mouse [18] similarly reported that *pax6* is not required for GABA amacrine cells, but is required for glycine amacrine cells. Thus, amacrine cells are not specified as a class by a single genetic program; instead, progenitors of different amacrine cell subtypes are differentially responsive to Rx1 and Pax6 at different developmental times (Figure 5). It should be mentioned that the reduction of certain amacrine cell types is not due to cell death or a change in fate to other tissues, but rather to effects on proliferation and differential maintenance of stem and progenitor marker genes [Zaghloul and Moody, unpublished].

### Timing of the effects

The timing of differentiation of the different retinal cell types is well conserved in vertebrates. Ganglion cells and horizontal cells are the first cells to be born followed by cone photoreceptors, amacrine cells, rod photoreceptors, bipolar cells, and finally Müller glia [4]. The temporal order in which retinal cell types are born is consistently maintained in vertebrates suggesting a role in cell type determination. In most animals, amacrine cells exit the terminal cell cycle over a broad temporal window. For example, in *Xenopus*, amacrine cells are born predominantly between stage 27 and 37 [48]. Our data indicate that the effects of altering Rx1 and Pax6 levels/activity on amacrine cells depend upon the time at which the changes are initiated (Figure 5); we assume that these changes in protein levels/activity are maintained throughout retinogenesis after hormone treatment, based on the long-lived activity of similar GR constructs in other studies [24,25,31,32], but we do not have antibodies that recognize the fusion proteins to prove this point directly. With this caveat in mind, although DA amacrine cell production was equally affected by increased Rx1 or Pax6 levels begun at either eye field stage, the NPY and GABA phenotypes showed distinct temporal effects (Figure 5). GABA cells were strongly repressed by Rx1 at the early stage and minimally affected at the late stage. NPY cells were repressed by Rx1 only at the late stage, and were repressed by Pax6 only at the early stage. Since a few studies in the rat retina suggest that amacrine subtypes may have slightly different birthdates [49,50], it is possible that altering Rx1 and Pax6, both of which have been implicated in controlling proliferation [9,51], at different eye field stages may differentially affect amacrine subtype birthdates.

The different temporal effects we observed also may be caused by the changing signaling environment in which cells reside as the retina develops [37]. For example, retinal progenitor cells that produce amacrine cells give rise to different subsets of progeny depending upon the embryonic stage at which the cells are isolated [52]. Temporal changes in NeuroD activity, modulated by glycogen synthase kinase-3 activity, also can affect cell fates [53]. Since Rx1 and Pax6 may hold cells in an immature, multi-potent state [9,51], temporally altering their levels of activity may regulate the competence of the cells to respond to changing environmental cues and thereby affect the production of different amacrine subtypes.

In addition, Rx1 and Pax6 likely interact in a time-dependent manner with other eye field transcription factors, such as Eye-gone [54], Six3 [55-59], Six6 [60-62] and Tll [63]. A recent study shows that in *Xenopus*, as proposed in *Drosophila* [64], there is a self-regulating feedback network of these factors that specifies the eye field to be the repository of retinal stem cells [5]. These authors demonstrated that the expression of a cocktail of eye field factors (ET, Lhx2, Pax6, Rx1, Six3, Six6, Tll) induced an ectopic eye field at high frequency. They further demonstrate that ET functions upstream of Rx1, which is upstream of Pax6, and that Tll and Six6 function later in the network. Thus, eye field transcription factors regulate each other and themselves over developmental time to provide the appropriate transcriptional environment for the expression of a retinal fate. Altering Rx1 and Pax6 levels/activity at different developmental times is likely to differentially impact elements in this transcriptional network. Thus, it will be important to study the effects of altering Rx1, Pax6 and the other eye field transcription factors in combination to fully understand the roles of these proteins in amacrine cell production.

The cross- and auto-regulatory interactions between the transcription factors in the retinal transcriptional network may explain two observations presented herein that at first glance seem contradictory. First, the loss-of-function phenotypes for both Rx1 and Pax6 are very similar for each of the three amacrine subtypes. This is not surprising if each gene regulates the other in a feedback loop, as proposed elsewhere [5]. Second, DA amacrine cells are significantly reduced by both increased and decreased levels/activity of Rx1 and Pax6, albeit the reduction is much less severe for the loss-of-function condition. Perhaps the DA amacrine cell phenotype is quite sensitive to the cross- and auto-regulatory part of the early transcriptional network. Continued work on exactly how these factors regulate each other will be critical for understanding how they influence the determination of the many subtypes of amacrine cells.
